# Non-coding RNA may be associated with cytoplasmic male sterility in *Silene vulgaris*

**DOI:** 10.1093/jxb/erx057

**Published:** 2017-03-29

**Authors:** James D. Stone, Pavla Koloušková, Daniel B. Sloan, Helena Štorchová

**Affiliations:** 1 Institute of Experimental Botany v.v.i, Academy of Sciences of the Czech Republic, Rozvojová 263, Prague, 16502Czech Republic; 2 Institute of Botany v.v.i, Academy of Sciences of the Czech Republic, Průhonice, Central Bohemia, 25243Czech Republic; 3 Colorado State University, Department of Biology, Fort Collins, CO 80523, USA

**Keywords:** Cytoplasmic male sterility, editing, mitochondrion, non-coding RNA, *Silene vulgaris*, splicing, transcriptome.

## Abstract

Cytoplasmic male sterility (CMS) is a widespread phenomenon in flowering plants caused by mitochondrial (mt) genes. CMS genes typically encode novel proteins that interfere with mt functions and can be silenced by nuclear fertility-restorer genes. Although the molecular basis of CMS is well established in a number of crop systems, our understanding of it in natural populations is far more limited. To identify CMS genes in a gynodioecious plant, *Silene vulgaris*, we constructed mt transcriptomes and compared transcript levels and RNA editing patterns in floral bud tissue from female and hermaphrodite full siblings. The transcriptomes from female and hermaphrodite individuals were very similar overall with respect to variation in levels of transcript abundance across the genome, the extent of RNA editing, and the order in which RNA editing and intron splicing events occurred. We found only a single genomic region that was highly overexpressed and differentially edited in females relative to hermaphrodites. This region is not located near any other transcribed elements and lacks an open-reading frame (ORF) of even moderate size. To our knowledge, this transcript would represent the first non-coding mt RNA associated with CMS in plants and is, therefore, an important target for future functional validation studies.

## Highlight

A comparison of mitochondrial transcript levels and RNA editing between females and hermaphrodites of *Silene vulgaris* suggests that mitochondrial non-coding RNA is putatively associated with cytoplasmic male sterility.

## Introduction

Unlike the small and gene-dense mitochondrial (mt) genomes of animals, the mt genomes of flowering plants are large and highly variable in size (66 kb to 11.3 Mb; Alverson *et al.*, 2010, 2011; Sloan *et al.*, 2012*a*; Skippington *et al.*, 2015). These genomes contain numerous regions of unknown origin, as well as DNA transferred intracellularly from the nucleus or plastids or transferred horizontally from different species (Rice *et al.*, 2013).

These large angiosperm mt genomes undergo frequent recombination, resulting in extensive genomic rearrangements (Arrieta-Montiel and Mackenzie, 2011). Recombination across short repeats can create chimeric open-reading frames (ORFs) that are occasionally transcribed and translated. Cytoplasmic male sterility (CMS) can result when such chimeric mt genes interfere with mt function and lead to the production of aborted anthers or inviable pollen (Horn *et al.*, 2014; Touzet and Meyer, 2014). In some cases, nuclear fertility-restorer (*Rf*) genes may inhibit CMS gene expression and restore pollen production (Hu *et al.*, 2014). CMS thus represents a mitochondrial–nuclear interaction and has been intensively studied to understand plant mt genome evolution (Ingvarsson and Taylor, 2002; Stadler and Delph, 2002; Darracq *et al.*, 2011; Fujii *et al.*, 2011*a*; Luo *et al.*, 2013). CMS has also been widely exploited in crops to produce hybrids with high yields, owing to heterosis (Chen and Liu, 2014; Huang *et al.*, 2014).

Diverse mitochondrial and nuclear genes are involved in CMS (Horn *et al.*, 2014; Touzet and Meyer, 2014). Chimeric mitochondrial CMS genes often comprise pieces of essential genes, including those encoding ATP synthase or cytochrome *c* oxidase subunits (Sabar *et al.*, 2003; Hanson and Bentolila, 2004; Gillman *et al.*, 2007; Kazama *et al.*, 2008). Completely unknown or unrecognizable ORFs also frequently contribute to these chimeric genes (Kim *et al.*, 2007; Duroc *et al.*, 2009). The nuclear *Rf* genes suppress the effects of CMS genes either by mRNA cleavage/degradation (Sabar *et al.*, 2003; Kazama *et al.*, 2008; Wang *et al.*, 2013) or by inhibition at the post-translational level (Luo *et al.*, 2013). Additionally, post-transcriptional modification through RNA editing (Hammani and Giegé, 2014) of *CMS* mitochondrial transcripts can be involved in fertility restoration. The unedited *orfB* gene, for example, was found to underlie male sterility in the wild abortive (WA) cytoplasm of rice (Chakraborty *et al.*, 2015). Furthermore, complex mitochondrial–nuclear interactions related to changes in amino acid sequence might be associated with CMS. For example, the CMS-G mt genome in sugar beet harbors mutations in the *cox1*, *cox2*, and *cox3* genes but does not contain any candidate CMS gene or ORF (Darracq *et al.*, 2011). The COX proteins were shown to be altered and the activity of cytochrome *c* oxidase reduced in male sterile plants. Compensatory mutations in the nuclear genes encoding subunits of complex IV were proposed to restore male fertility in sugar beet CMS-G (Darracq *et al.*, 2011).

The colorful mosaic of molecular mechanisms underlying CMS in agricultural plants probably represents only a tiny fraction of the overall variety of male sterilizing factors in angiosperms. Gynodioecy, a widespread plant breeding system characterized by the co-occurrence of female (F) and hermaphroditic (H) individuals within populations, is often associated with CMS (McCauley and Olson, 2008). Because of its importance in crop breeding, the genetic background of CMS has thus far been investigated primarily in agricultural plants, and very few CMS systems have been characterized at the molecular level in natural populations (Case and Willis, 2008; Darracq *et al.*, 2011; Mower *et al.*, 2012; Case *et al.*, 2016).

One of the most thoroughly explored examples of CMS in natural populations is in bladder campion (*Silene vulgaris*), a model system for the investigation of gynodioecy (Bernasconi *et al.*, 2009), used in numerous population genetic studies (e.g. Olson and McCauley, 2002; Štorchová and Olson, 2004; Sanderson *et al.*, 2016). An unprecedented level of intraspecific diversity has been revealed in the complete mt genome sequences of four haplotypes of this species (Sloan *et al.*, 2012*b*). They differed not only in the sequence and structure of intergenic regions but also in their gene content. The mt genome of *S. vulgaris* undergoes frequent recombination and is fragmented into several circular-mapping chromosomes, with the number of chromosomes varying among haplotypes (Sloan *et al.*, 2012*b*).

It has been proposed that elevated levels of mtDNA polymorphism in some *Silene* species (including *S. vulgaris*) are maintained by balancing selection acting on CMS genes (Stadler and Delph, 2002; Houliston and Olson, 2006; Touzet and Delph, 2009). Several candidate CMS genes were identified in the complete mt genomic sequences of *S. vulgaris* (Sloan *et al.*, 2012*b*), including the *Bobt* gene which was differentially expressed between F and H plants (Štorchová *et al.*, 2012).

Recent improvements in high-throughput sequencing methods greatly facilitate the discovery of CMS genes, which can be predicted in whole-genome sequences based on their chimeric nature or as unknown ORFs (Grewe *et al.*, 2014). Differentially expressed genes and differentially edited RNAs associated with CMS may be identified by comparing mt transcriptomes of male-sterile and male-fertile plants (Stone and Štorchová, 2015).

In this study, we used RNA-seq to comprehensively investigate mt gene expression in *S. vulgaris*. We chose the KOV haplotype because no chimeric CMS candidates (chimeric ORF >50 aa) were found in its mt genome (Sloan *et al.*, 2012*b*). We compared transcript abundances and editing extent between F and H siblings with the aim of identifying features associated with CMS. We also performed an analysis to determine the order in which RNA editing and intron splicing events occur. Although F and H transcriptomes were very similar overall, our analyses revealed a single genomic region that was highly overexpressed and differentially edited in Fs relative to Hs. This region lacks an ORF of even moderate length. If a causal role in male sterility were confirmed for this transcript, it would represent the first case, to our knowledge, of a non-coding mt RNA associated with CMS in plants.

## Methods

### Plant material

*Silene vulgaris* KOV haplotype was collected in the Czech Republic in Kovary Meadows (Elansary *et al.*, 2010). A single F plant was pollinated by an H plant of the same haplotype originating from a different mother in 2012. The progeny were cultivated in the Institute of Experimental Botany greenhouse under supplemental lighting (16/8 h light/ dark) in pots filled with perlite, vermiculite, and coconut coir (1:1:1), and fertilized 2–3 times per week depending on the season. The plants were cut back multiple times to prevent self-seeding. Three F and three H full-sib individuals were selected and tested for homoplasmy by amplifying, cloning, and sequencing the highly polymorphic *atp1* gene, using the pGEM T-easy vector (Promega, WI, USA). The sequences of all 20 clones from each individual were identical.

### RNA and DNA extraction

Flower buds (<3 mm) from each individual were collected and flash-frozen in liquid nitrogen. Total RNA (>100 nt) was extracted with an RNeasy Plant Mini Kit (Qiagen, Germany). Additional flower buds were collected at the same time and used to isolate total DNA with a DNeasy Plant Mini Kit (Qiagen) for the estimation of gene copy number. RNA was treated with DNase I, using a DNA-free kit (Ambion, Foster City, TX, USA). If necessary, the DNase treatment was repeated twice to eliminate any traces of genomic DNA. A 10-µg sample of RNA from each individual was dried in GenTegra tubes (GenTegra, Pleasanton, CA, USA) according to the manufacturer’s instructions, and sent to the USC Epigenome Center (CA, USA). Total RNA and DNA were also extracted from leaves, lateral roots, and pollen by the same method for transcript and gene copy number quantification. Pollen DNA was obtained in very small concentrations that prevented reliable qPCR measurements, so only transcript levels were determined for pollen.

### Transcript level and gene copy number estimation

Complementary DNA (cDNA) was synthesized using Transcriptor HF Reverse Transcriptase (Roche Applied Science, Mannheim, Germany) following the manufacturer’s instructions. Briefly, 300 ng of RNA and 1.2 nmol of random hexamers (total volume 11.4 μl) were heated for 10 min at 65 °C, chilled on ice, and mixed with Transcriptor buffer, 0.5 μl of Protector RNase Inhibitor, 2 μl of 10 mM dNTPs, 1 μl of 100 mM DTT, and 10 units of reverse transcriptase (RT) in a final volume of 20 μl. First-strand cDNA was synthesized at 29 °C for 10 min, then 48 °C for 60 min with final heating to deactivate reverse transcriptase at 85 °C for 5 min. Two independent RT reactions were carried out for each RNA sample.

Quantitative PCR was performed using Light Cycler 480 SYBR Green I Master on a LightCycler 480 instrument (Roche Applied Science). The reaction mixture contained 5 µl 2× MasterMix, primers in specific concentrations (see Supplementary Data Set S1F at *JXB* online), and 2.5 µl of 20× diluted first-strand cDNA in a total volume of 10 µl. Initial denaturation for 5 min at 95 °C was followed by 50 cycles of 10 s at 95 °C, 10 s at 60 °C (mt *rrn18* at 58 °C, nuclear 18S rRNA at 64 °C), and 15 s at 72 °C. Amplification efficiencies were estimated from calibration curves generated from a serial dilution of cDNA. The relative ratio of a target gene was calculated as *e*_r_^CPr^/*e*_t_^CPt^, where *e*_r_ and *e*_t_ correspond to the PCR efficiencies of the reference and target genes, respectively, and CPr and CPt correspond to crossing points (calculated as the maximum of the second derivate of the fluorescence curve). A negative control was performed using RNA instead of cDNA as a template in the reaction mixture. Transcript levels were normalized with *S. vulgaris* mt *rrn18*. Each cDNA sample was measured twice, and means and standard deviations (SD) were calculated from four values (2 cDNAs × 2 measurements).

Gene copy number was estimated by qPCR in the same way as transcript abundance. The intragenomic ratio of mt gene copy numbers was assessed relative to the mt *rrn18* gene. The ratio between mt DNA and nuclear DNA was estimated relative to nuclear 18S rDNA. Each measurement was repeated four times.

### Illumina sequencing, read trimming, and quality filtering

Strand-specific cDNA libraries were prepared from total RNA after rRNA elimination using Ribo-Zero Plant Leaf rRNA Removal Kit (Illumina, San Diego, CA).). Mapped reads indicated a mean fragment length of 227 nt with a SD of 118 nt. Illumina HiSeq 2000 sequencing generated paired-end reads (2 × 75 cycles). The reads were trimmed using Trimmomatic 0.32 (Bolger *et al.*, 2014) in paired-end mode, which removed low-quality ends and any sequence after the average quality of a four-base window dropped below 20. Reads with a post-trim length of less than 48 were removed. A total of 82.3% of read pairs passed filtering, and an additional 13.8% of pairs had a single read pass. The mean post-trimming read length was 74.1 bp.

### Bioinformatic analyses

Initial alignment was performed using GSNAP v. 2014-12-23 (Wu and Nacu, 2010) in paired-end mode and default settings otherwise. GSNAP splice site prediction agreed with the annotated exons and introns in the KOV mt genome (Sloan *et al.*, 2012*b*), and no chimeric transcript was detected in any RNA sample. A new alignment was performed using known splice junctions as *a priori* information for GSNAP.

To reveal additional differentially expressed transcripts not discovered by read mapping constrained to the genomic reference, we performed *de novo* assembly using only reads that mapped to the mt genome. Trinity 2.2.0 (Grabherr *et al.*, 2011) was used to assemble these paired-end, strand-specific reads. These mt-mapped reads were then aligned to the *de novo* transcriptome, using bowtie2 (Langmead and Salzberg, 2012), as implemented in Trinity. RSEM and edgeR (Robinson *et al.*, 2010) were used to allocate reads, estimate transcript abundances, and identify differentially expressed transcripts, according to Trinity’s recommended workflow. Detailed bioinformatic procedures are described in Supplementary Method S1.

### Accession number

Transcriptomic data from this article can be found in the GenBank Short Read Archive under the accession number PRJNA321915.

## Results

### Deep sequencing and read mapping

We produced RNA-seq data from total RNA extracted from the flower buds of three F and three H *S. vulgaris* individuals. After rRNA elimination and RNA fragmentation, all samples were sequenced in a single Illumina run, generating approximately 33.3M read pairs per sample. On average, 82% of read pairs passed quality filtering (27.4M per sample). An additional 4.6M pairs had only a single read pass, with 75% of these being the first read. These reads were then mapped against the mt genome of *S. vulgaris* KOV, which consists of six chromosomes (GenBank accession numbers JQ771300–JQ771305). An average of 2.92M pairs per sample (10.7% of read pairs after quality filtering) mapped to the mt genome. The mapping yield was reduced to 2.40M pairs per sample (8.8%) when mt regions with high plastid similarity were excluded. The strand-specific library preparation was generally effective, with coding regions showing the expected pattern of sense-strand coverage dominating antisense-strand coverage. This was not absolute, however, as several reads mapped to the antisense strand but showed editing that is only possible on the sense strand. Assuming G-to-A edits do not occur, a small fraction of reads, varying from less than 0.5% to 10%, appears to have been attributed to the incorrect strand.

### Depth of coverage (DOC) of mt genes and genomic regions

We estimated read coverage of protein-coding genes, ORFs longer than 300 bp, and intergenic transcribed regions (‘transcription islands’). We also recorded DOC for rRNA and tRNA features, but their values are not representative: rRNA was depleted before the construction of cDNA libraries to enrich organellar mRNA, while small RNAs (< ~100 nt), including tRNAs, were lost in the course of column-based RNA extraction.

**Fig. 1.**
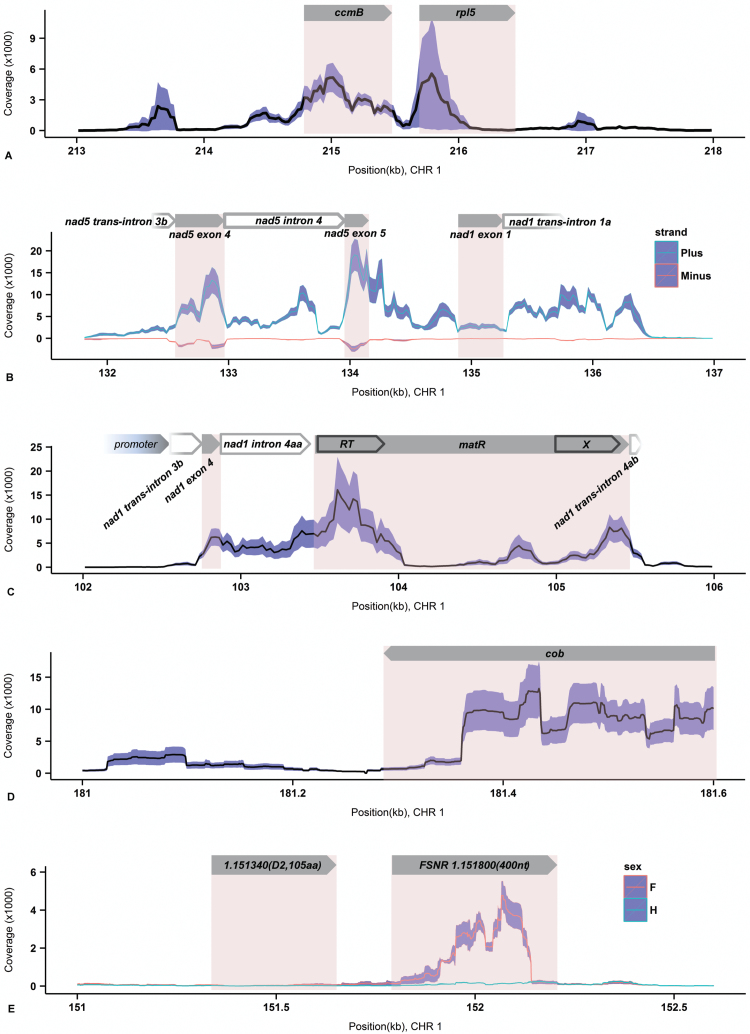
RNA-seq read coverages for genes and intergenic regions. Coverage was calculated based on a sliding window with a size of 75 bp and a step size of 25 bp for (A–C), and on a single base resolution for (D) and (E). Mean depth of coverage (DOC) values across individuals are given by the lines of the plots, while standard deviations in DOC are given by the blue bands. Values in (A–D) are for all six individuals examined, and in (E) they are split into female (*n*=3) and hermaphrodite (*n*=3). (A) The *ccmB* and *rpl5* genes. (B) Exons and introns of the *nad5* and *nad1* genes. Antisense coverage is shown with negative *y*-values. (C) The *matR* gene with a ‘hole’ in the middle of the coding region. (D) The *cob* gene with transcripts lacking a stop codon. (E) *FSNR*, which is highly expressed in females and not expressed above background levels in hermaphrodites. The size of this region is defined according to the DOC threshold for ‘transcription islands’.

DOC varied roughly 10-fold among protein-coding genes, with most between 5000 and 50 000 transcripts per million (TPM) (Wagner *et al.*, 2012). The most highly expressed feature was *atp1*, whereas *matR*, *ccmB*, *ccmFc*, and *ccmFn* exhibited the lowest DOC (see Supplementary Data Set S1A). Coverage values were consistent across all six individuals, with standard deviations generally less than 30%. The most prominent exception was *rpl5* (Fig. 1A), which exhibited low levels of coverage that varied approximately 10-fold among individuals.

Six mt genes of *S. vulgaris* contain type II introns, and three of them (*nad1*, *nad2*, *nad5*) generate trans-spliced transcripts. DOC of individual exons of the same gene varied substantially (Supplementary Data Set S1A). For example, the *nad7* exon 4 was covered only ~30% as deeply as adjacent exons. Introns were, in general, covered less than the neighboring exons, but their DOC still reached values typical for protein-coding genes. A few introns, such as *nad1 trans*-intron 1.2a (‘a’ denotes the 5′ portion of the *trans*-spliced intron) (Fig. 1B) or *nad2* intron 3.4, exhibited a similar or higher level of coverage than adjacent exons. High coverage of some introns may reflect their independent existence after splicing out of primary transcripts.

Most *trans*-spliced introns are located far from transcribed regions and therefore must utilize their own promoters. However, *nad2* exons 3–5 may be co-transcribed with the adjacent *trnY* gene. In addition, *nad1* exons 2–3 seem to be co-transcribed with the *rps13-b* pseudogene, which represents only the 3′ end of *rps13*. The 5′ end of this gene, including the promoter (*rps13-a*), was translocated to chromosome 3 in the *S. vulgaris* KOV haplotype. Therefore, the *rps13-b* pseudogene appears to have acquired a novel promoter that is now associated with the transcription of *nad1* exons 2–3, which may explain the relatively high DOC (>5000 TPM) for the *rps13-b* pseudogene compared to the DOC for the *rps13-a* pseudogene (~200 TPM) (Supplementary Data Set S1A).

Individual exons and mono-cistronic genes generally showed only moderate variation in DOC, but *matR* represented a dramatic exception. The internal part of this gene had much lower coverage than the first and last thirds of the coding sequence, which correspond to the reverse transcriptase (RT) and and maturase (X) domains, respectively (Fig. 1C). In addition, a sudden drop in coverage before the first in-frame stop codon was observed in *atp1* (see Supplementary Fig. S1A), *nad6* (Fig. S1B), *ccmC* (Fig. S1C), and *cob* (Fig. 1D), suggesting many of these transcripts lack stop codons.

We analyzed the coverage of 27 ORFs longer than 300 bp (100 aa) that were previously identified in the *S. vulgaris* KOV mt genome (Sloan *et al.*, 2012*b*). Only ORFs within introns or in close proximity to coding sequences were covered at a level comparable to protein-coding genes (see Supplementary Data Set S1B). We identified ‘transcription islands’ in intergenic regions, as defined using the distributions of coverages in annotated and non-annotated areas (see Methods). Several such islands were located in the vicinity of genes, and their transcription might not be independent (Supplementary Data Set S1C). There were, however, 15 transcription islands with DOC reaching the levels of protein-coding genes and located hundreds or thousands of bp from the nearest transcribed features (Supplementary Data Set S1C).

### Differential expression between F and H individuals

In general, DOC was similar between F and H individuals and consistent across biological replicates. There was only one feature strongly differentially expressed between F and H, located on chromosome 1 between the positions 151 800 and 152 200 (Fig. 1E). This region had ~15-fold higher DOC in F than in H individuals, with a standard deviation less than 15% in each gender. The longest ORF in this region was a mere 18 bp. We refer to this feature as *Female-Specific Noncoding RNA* (*FSNR*). We found no similar sequence in GenBank by BLASTN or BLASTP search, except for a nearly identical sequence in the mt genome of *S. vulgaris* MTV (JQ771306). *FSNR* must be transcribed from its own promoter because no transcribed feature occurs in its vicinity. The CGTATAA motif found at positions 151 759–151 765 is a good candidate for the *FSNR* promoter (Kühn *et al.*, 2005).

Protein-coding genes were expressed similarly in both genders. A modest exception, *ccmFn*, had ~2.3-fold higher DOC in H than in F individuals (Supplementary Data Set S1A). In contrast, *ccmFc* had slightly lower DOC in H than in F individuals. This resulted in a roughly 3-fold excess of *ccmFc* over *ccmFn* in F individuals, whereas the two transcripts were roughly equimolar in H individuals.

### Experimental estimation of mt gene expression and copy number

**Fig. 2.**
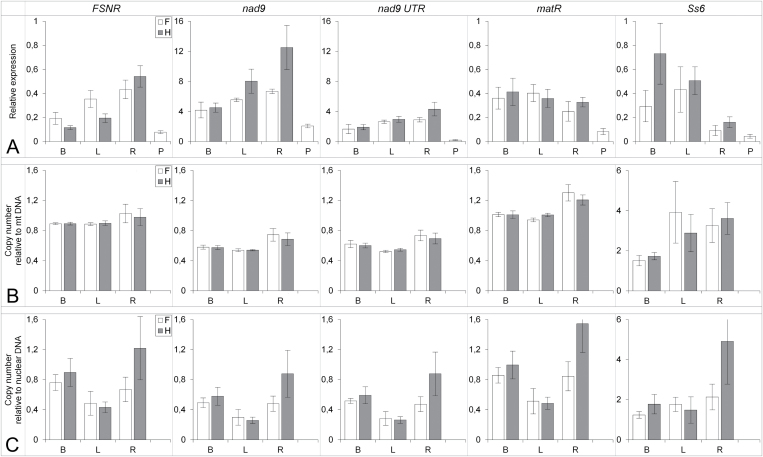
Relative expression and copy number of the selected genes and regions in mt DNA of *S. vulgaris*. (A) Gene expression relative to mt 18S rRNA. (B) Copy number relative to mt 18S rDNA. (C) Copy number relative to nuclear 18S rDNA. White bars are females (F), gray bars are hermaphrodites (H). B, flower buds; L, leaves; R, roots; P, pollen. Values are means ±SD, *n*=6.

We developed reverse-transcriptase quantitative PCR (RT qPCR) assays to examine the transcript levels and copy numbers of selected mt genes or genomic regions. We analyzed RNA and DNA extracted from flower buds, leaves, roots, and pollen of the same six individuals used for RNA-seq and six additional full-sibs. *FSNR* showed significantly higher expression in F than in H in flower buds and leaves (Fig. 2A), although the difference was only 2-fold, much lower than the 14-fold difference estimated from RNA-seq data. We also used qPCR to measure *FSNR* expression in flower buds of three additional F and three additional H individuals from the same cross, as well as from five F and five H plants from two independent crosses. We found 2- to 3-fold differences, which were similar to the qPCR values detected in the plants used for RNA-seq analyses. The simplest explanation for the discrepancy between qPCR and RNA-seq DOC would be trace contamination with genomic DNA. We excluded this possibility noting the absence of PCR amplification when RNA was used as a template. The preparation of cDNA libraries for RT qPCR and Illumina sequencing follows different protocols, which might account for the observed quantitative inconsistency of the two methods. *FSNR* transcript levels in roots were comparable between the sexes (Fig. 2A).

The *FSNR* copy number in genomic DNA measured against mt *rrn18* was close to 1 across all of the samples, confirming equimolar ratios among the mt genes. In contrast, the estimated copy number of *FSNR* relative to nuclear 18S rDNA was lower in leaves compared to other tissue types, a pattern that was also observed for other mt genes and regions. This mt copy number reduction suggests a lower mt DNA content in the leaves, relative to nuclear DNA (Fig. 2C).

We selected the coding sequence (CDS) and 5′ untranslated region (UTR) from *nad9* as an example of a highly expressed mt gene and from *matR* as an example of a low-expression gene. The RT qPCR results were in agreement with DOC for each of the respective genes. The *nad9* transcript levels were the highest, and *matR* transcript levels were the lowest with no significant differences between H and F individuals. Interestingly, all four tested genic regions showed non-significant variation in the ratio of mt DNA to nuclear DNA between F and H plants. The ratio was slightly higher in H flower buds and in H roots.

Finally, we chose an intergenic region with variable DOC for RT qPCR validation. The region between positions 1–400 of the smallest chromosome 6 (called *Start of the smallest 6* or *Ss6*) displayed moderate DOC in some individuals and nearly zero coverage in others with no clear association with gender. Chromosome 6 is highly conserved among the four sequenced *S. vulgaris* mt genomes (Sloan *et al.*, 2012*a*), which suggests its functional importance, despite the absence of any gene. Transcription of this chromosome further indicates its functionality.

RT qPCR measurements showed variable transcription of the *Ss6* region, higher in flower buds and leaves than in roots or pollen. Unlike other mt regions with nearly equimolar ratios, the *Ss6* copy number was 2- to 5-fold higher than mt *rrn18*. Leaves and roots contained more copies of this region (and probably of the entire chromosome 6) than flower buds. When the variation in *Ss6* copy number is taken into account, we may conclude that *Ss6* is transcribed highly in flower buds, less so in leaves, and very little in roots, with large differences among individuals (Fig. 2B).

### RNA editing differences between females and hermaphrodites

We examined C-to-U RNA editing throughout the *S. vulgaris* KOV mt transcriptome. We identified 377 RNA editing sites (see Supplementary Data Set S1D, Fig. S2) that met minimum coverage and quality thresholds. We verified 31 additional sites that were either undetected or did not meet quality thresholds by revisiting sites edited in other taxa. Thus, we confirmed a total of 408 well-supported, single-copy editing sites in the *S. vulgaris* KOV mt transcriptome.

**Fig. 3.**
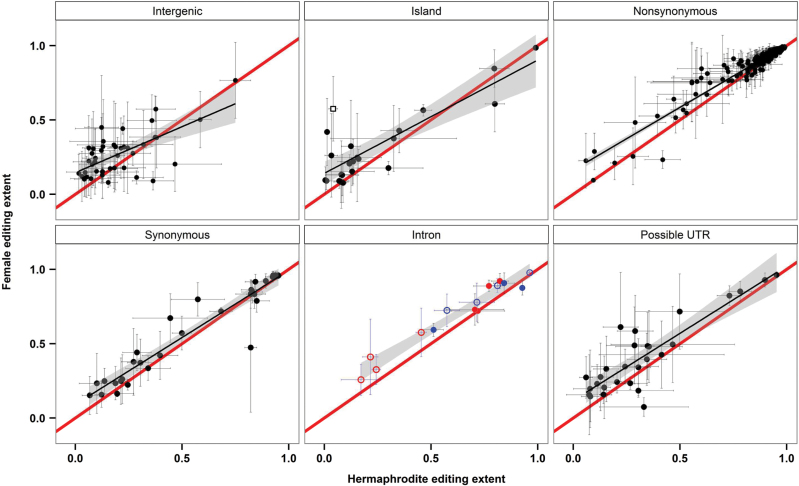
RNA editing extent in *Silene vulgaris* mitochondria, Female vs. Hermaphrodite, organized by editing site location. Mean values (±SD) for females (*n*=3) and hermaphrodites (*n*=3) are plotted. A line representing equal editing extent for both genders is plotted as *y*=*x*, in red. A 95% confidence band for the observed data is given in gray within each plot. Within the intron panel, sites predicted to stabilize intron structure are indicated with filled circles, while sites not known to influence intron stability are depicted with open circles. In addition, within the intron panel, sites within *cis*-introns are colored red, while *trans*-intron sites are colored blue. Lastly, within the island panel, the editing site located in the *FSNR* is indicated with an open square.

Editing was generally conserved among individuals and consistent between sexes (Fig. 3, Supplementary Data Set S1D). One notable exception was the partially edited site within the *FSNR* (position 152 031; Supplementary Data Set S1D), which was nearly unedited in H individuals and more than 50% edited in F individuals. This suggests that not only is the *FSNR* transcript differentially expressed between the sexes, but that a corresponding editing factor is also differentially expressed.

### Distribution of editing sites

There were 298 unique editing sites in protein-coding regions (including *rpo1* and excluding introns), 264 of which result in an amino acid change (Table 1). The *ccm* genes tended to show high editing-site density, whereas *cox1* (no editing sites), *atp8* (one editing site), and *atp1* (three editing sites) had the lowest densities.

**Table 1.** Editing sites in the mt protein genes of three *Silene* species

	Length (bp)	*S. vulgaris*	*S. latifolia*	*S. noctiflora*
	Complex I			
*nad1*	978	19(2)	19(2)	11(1)
*nad2*	1467	**23**(4)	21(3)	18(3)
*nad3*	357	**8(1**)	8(1)	5(1)
*nad4*	1488	16(0)	16(0)	11(0)
*nad4L*	303	**10**(1)	9(0)	6(1)
*nad5*	2019	18(0)	18(0)	15(0)
*nad6*	570	**11(2**)	10(2)	6(0)
*nad7*	1173	19(1)	19(1)	9(1)
*nad9*	579	**6(1**)	5(0)	1(0)
	Complex III			
*cob*	1164	9(1)	9(1)	6(0)
	Complex IV			
*cox1*	1572	0(0)	0(0)	0(0)
*cox2*	777	**4(0**)	3(0)	2(0)
*cox3*	798	1(0)	1(0)	1(0)
	Complex V			
*atp1*	1509	**3(0**)	3(0)	0(0)
*atp4*	555	**13(1**)	11(1)	6(0)
*atp6*	720	11(1)	11(1)	7(0)
*atp8*	492	**1(0**)	2(1)	2(1)
*atp9*	225	**2**(**0**)	4(1)	1(0)
	Cytochrome C biogenesis			
*ccmB*	621	**30(6**)	27(4)	19(2)
*ccmC*	666	**24(4**)	23(3)	15(1)
*ccmFc*	1341	**14(3**)	12(1)	8(1)
*ccmFn*	1743	**23(2**)	22(0)	16(2)
	Ribosomal			
*rpl2*	1050	NP	NP	NP
*rpl5*	558	6(0)	6(0)	4(0)
*rpl16*	405	NP	NP	NP
*rps3*	1050	NP	4(0)	3(0)
*rps4*	1089	NP	NP	NP
*rps7*	447	NP	NP	NP
*rps12*	378	NP	NP	NP
*rps13*	351	0(0)	1(0)	0(0)
	Other			
*matR*	1986	8(1)	8(1)	6(1)
*mttB*	753	**18(3**)	15(0)	11(1)
Total		297(34)	287(23)	189(16)

The number of synonymous editing sites is in parentheses. NP indicates the absence of a functional copy in the mt genome. The numbers of editing sites that are different between *S. vulgaris* and *S. latifolia* are in bold.

We found 15 editing sites in type II introns, nine of them located in *cis*-spliced introns and six in *trans*-spliced introns. Six editing sites are located in the stems of domains IV, V, or VI and may contribute to the stability of the RNA’s predicted secondary structure. We compared intron edits in *S. vulgaris* with editing sites reported in four other species – two with comprehensive transcriptomes (*S. noctiflora* and tobacco) and two with partial evidence from selected genes (*Oenothera* and wheat; Supplementary Table S1). The number of intron editing sites in *S. vulgaris* is comparable to the 12 detected in tobacco (Grimes *et al.*, 2014) and in *S. noctiflora* (Wu *et al.*, 2015*a*). The editing site in domain I of *nad2 trans*-spliced intron 2.3 was the only intron editing site observed in all five species examined. Seven editing sites occurring in at least one species were absent in both *S. vulgaris* and *S. noctiflora*. Five of these were edited in tobacco. Finally, we detected 93 editing sites located in intergenic regions (including ORFs).

### Frequency of partial editing

The use of RNA-seq data allowed us to quantify the proportion of transcripts that were edited at a given site. Non-synonymous sites within CDS were edited to the highest extent, followed by sites stabilizing the secondary structure of group II introns. Synonymous sites were generally only partially edited, though still more so than ORF and intergenic sites (Fig. 3). Very few nearly-complete editing events were observed in intergenic space, with the majority showing less than 50% editing. Four intron sites were edited at a frequency of less than 50%, all of which were in *cis*-spliced introns. All identified *trans*-spliced intron edits occurred at a frequency of greater than 50%. None of the four intron sites edited at a frequency of less than 50% in *S. vulgaris* were found in *S. noctiflora*, *Oenothera*, tobacco, or wheat. In contrast, highly edited intron sites (with one exception) were observed in the homologous position in at least one additional species. The extent of editing at each site in intergenic regions and transcription islands was much lower than in protein-coding genes (Fig. 3). Only five of these sites were more than 50% edited and only one (chromosome 3, position 20 896) showed nearly-complete editing in all samples.

### The order of RNA editing and splicing

**Fig. 4.**
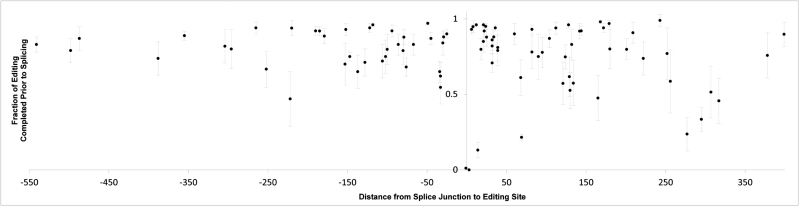
Editing achieved prior to splicing, as a fraction of post-splice editing extent for all sites with sufficient pre- and post-splice reads available (*n*=106 sites). The *x*-axis shows the distance from the nearest splice junction. Downstream positions are positive, upstream positions are negative. Data are mean values (±SD) across all six individuals.

Isolating reads derived from unspliced and spliced transcripts made it possible to compare the extent of editing in transcripts pre- and post-splicing for all sites neighboring splice junctions. At nearly every position, the majority of reads had been edited prior to splicing (Fig. 4). We also found that the extent of editing in mature (spliced) transcripts was greater than in pre-spliced transcripts for almost all sites (see Supplementary Data Set S1E).

At a few sites, editing and splicing happen only in a particular order (Fig. 4), primarily those immediately downstream from splice junctions (Table 2). In *nad7* exon 5 (3 bp downstream from a splice site), editing happened only after splicing. In a site in *nad1* exon 4 (14 bp downstream from a splice site), very limited editing is observed prior to splicing. In contrast, a site in *nad5* exon 2 (12 bp downstream from a splice junction) shows nearly complete editing prior to splicing, indicating the pre-splice *cis* motif is targeted by its editing factor. Comparisons of pre- and post-splice *cis* motifs preceding these two editing sites (Table 2) show differences of 5 or 4 nt, respectively. Although the post-splice editing site in *nad1* exon 4 is more distant from the splice junction than the pre-splice edit in *nad5* exon 2, the pre- and post-splice motifs differ more in *nad1* exon 4. An editing site located a single nucleotide upstream from a splice site in *nad7* exon 3 shows no editing prior to splicing, as was noted by Li-Pook-Than *et al.* (2007) in wheat. Interestingly, an editing site occurring six nucleotides downstream from a splice junction in *nad4* exon 3 was highly edited before splicing, in contrast with observations from wheat (Li-Pook-Than *et al.*, 2007).

**Table 2.** The extent of RNA editing in spliced (upper-case) and unspliced (lower-case) mitochondrial transcripts of six exons of *S. vulgaris* depending on the distance from the splice junction

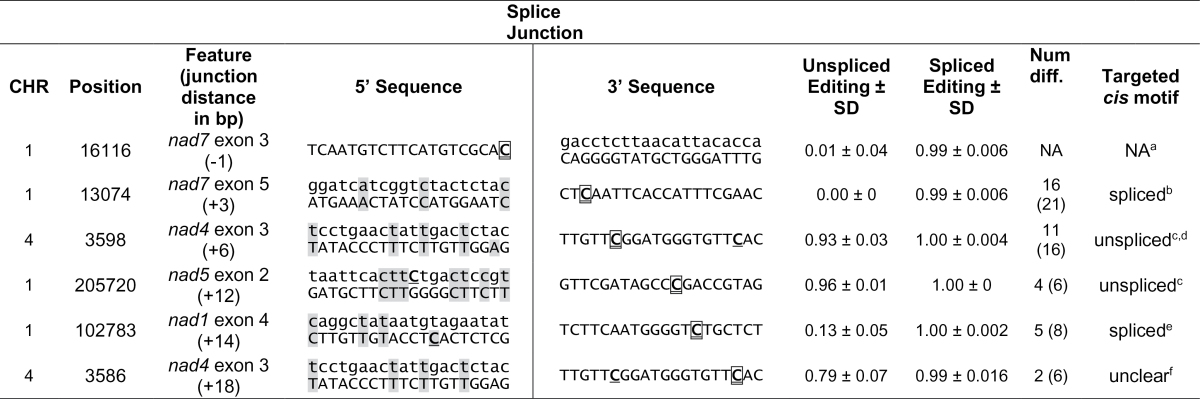

^a^ The 5′ cis-motif is unaltered by splicing. ^b^ Editing is not observed in unspliced transcripts. ^c^ Nearly-complete editing is observed prior to splicing, therefore the unspliced *cis*-motif must be sufficient for editing factor binding. ^d^ In contrast with *S. vulgaris*, this site appears only to be edited *after* splicing in wheat [*nad4* cd(+6); Li-Pook-Than *et al*., 2007]. ^e^ Very limited editing observed prior to splicing, probably due to 15/20 similar bases in the spliced and unspliced *cis* motifs. ^f^ Editing site is far enough from the splice junction that either *cis* motif may be sufficient for editing factor binding. Num. diff. – Number of differences between spliced and unspliced transcripts in 20 (25) nt upstream of the editing site. The exon sequence is shown in upper-case letters. Positions homologous between spliced and unspliced *cis*-motifs are shaded. 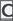 is the editing site under investigation, 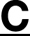 is another editing site in the depicted sequence.

**Fig. 5.**
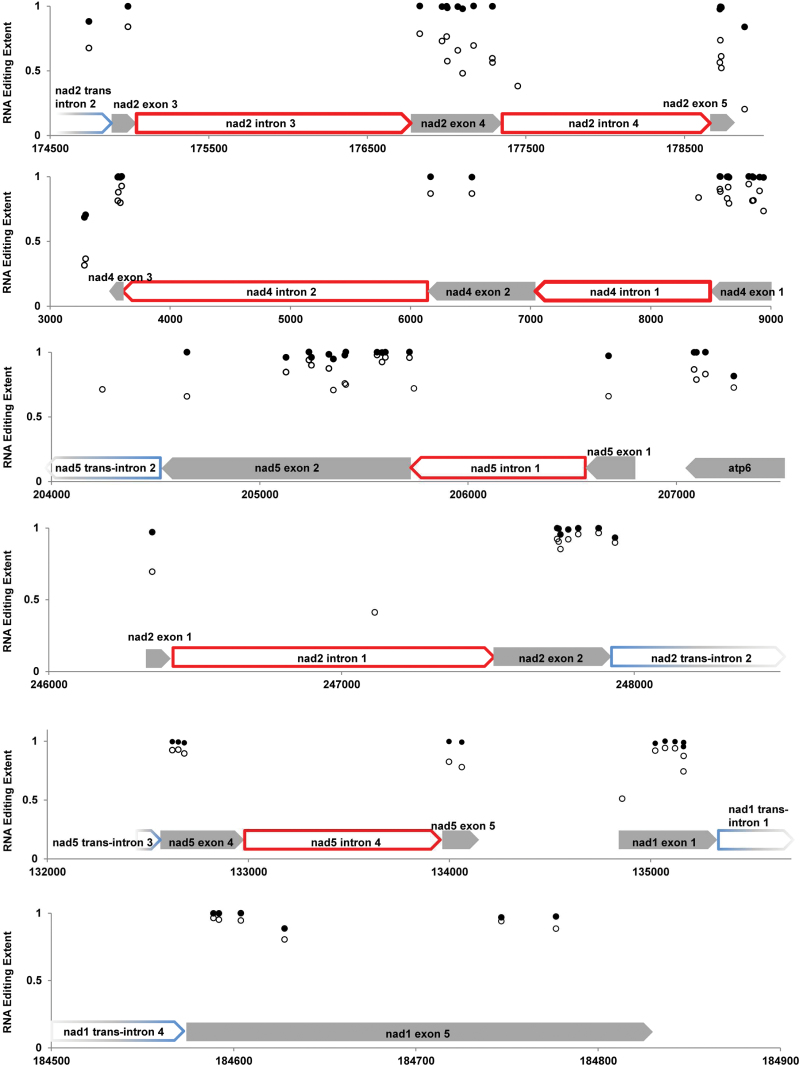
Editing extent before and after splicing for selected transcripts. Mean pre-splice edit extents are depicted on the *y*-axis with open circles and mean post-splice values are depicted with closed circles. Genomic coordinates are given on the *x*-axis. Exon boundaries are indicated above the *x*-axis with gray arrows and introns are depicted with open arrows. *Cis*-spliced introns are outlined in red and trans-spliced introns are outlined with a blue-to-gray fade, as distal trans-intron boundaries are not precisely known. In some cases, particularly within introns, only pre-splice editing extents could be calculated.

In summary, most editing reactions precede splicing reactions. The extent of editing prior to splicing varied from site to site within and between transcripts (Fig. 5). In contrast, variation among individuals was minor. There were no significant differences between F and H in the extent of pre-splice editing (see Supplementary Data Set S1E).

## Discussion

### *Potential association between a non-coding RNA and CMS in* S. vulgaris *KOV*

We have performed a comprehensive comparison of mt transcriptomes of female (F) and hermaphrodite (H) flower buds of *S. vulgaris* and found only one significantly differentially expressed feature: a transcription island located far from the nearest transcribed sequence. *FSNR* had ~15-fold higher coverage in F than H plants. The experimental estimation of the *FSNR* transcript levels by RT qPCR confirmed the differences between F and H in flower buds and leaves, albeit to a lesser extent than shown by DOC, and similar levels between the sexes in roots. The quantitative discrepancy between RT qPCR and RNA-seq data may be for either technical or biological reasons. We excluded contamination by genomic DNA experimentally. The protocol for cDNA preparation in RNA-seq, unlike RT qPCR, involves RNA fragmentation, which may account for the observed discrepancy.

There were numerous stop codons scattered in all three reading frames across 400 bp of the transcribed feature. The longest ORF was only 18 bp long, which seems to exclude the possibility that *FSNR* encodes a protein or peptide. While very short ORFs coding for functional small peptides have recently been identified in mammals and plants (Hellens *et al.*, 2016), they often occur in UTRs or are derived from known coding sequences. *FSNR* is not similar to any record in GenBank, except for a sequence in another mt genome of *S. vulgaris* MTV) (JQ771306). *FSNR* is not only differentially expressed, but it is also differentially edited, showing a much higher extent in F than in H. It is also the strongest case of differential editing between F and H individuals in our data set. High transcription and editing suggest a functional importance of *FSNR*, and possibly a role in CMS in *S. vulgaris*. The difference in expression levels between F and H individuals for *FSNR* contrasts with the overall similarity of mt transcription between F and H individuals for the rest of the genome. Minor differential expression was found in the *ccmFc* and *ccmFn* genes. Their transcripts were roughly equimolar in H plants, but a 3-fold excess of *ccmFc* over *ccmFn* was found in F plants. This subtle change may represent a consequence of CMS, but a causal link with male sterility cannot be excluded. The ORFs previously detected in the KOV genome (Sloan *et al.*, 2012*b*) were transcribed above background levels only in cases where they overlapped UTRs or introns (see Supplementary Data Set 1B). Low transcription makes it unlikely that any of these ORFs yield protein products that participate in CMS. In conclusion, our comparative study of mt transcriptomes rules out a more typical chimeric CMS gene and reveals *FSNR* as the strongest CMS candidate in *S. vulgaris* KOV. However, further functional characterization would be required to determine whether differential expression in this region is directly responsible for CMS, is part of a more complex mitochondrial–nuclear interaction leading to CMS, or is only a consequence of CMS.

It is difficult to imagine how this region may influence or impair mitochondrial functionality and pollen production. There is no report of mt non-coding RNA associated with CMS in plants and only a few studies recording non-coding RNA candidates in plant mitochondria (Dietrich *et al.*, 2015). Five non-messenger RNAs were described in *Arabidopsis thaliana* mitochondria based on a cDNA library survey (Marker *et al.*, 2002). Recent plant mt transcriptomic studies have confirmed that a high proportion of intergenic regions are transcribed, albeit mostly at low levels (Fujii *et al.*, 2011*b*; Grewe *et al.*, 2014; Grimes *et al.*, 2014). Wu *et al.* (2015*a*) conducted a comprehensive survey of small mt RNAs in *Silene noctiflora*. They mostly resulted from the degradation of long transcripts, but some candidates for functional small RNAs were also identified. The *FSNR* in *S. vulgaris* differs from most previously described plant mt non-coding mt RNAs by its large distance from transcribed genes or ORFs. It represents a true ‘transcription island’ within a genomic ocean.

As there is no sequence similarity between *FSNR* and the rest of mt genome, we cannot speculate about the possibility of sequence-specific interference, such as in the case of small interfering RNA. However, when we expand our search to ORFs in *FSNR* with non-canonical start codons UUG, AUA, or GUG, we find a 72-bp ORF beginning with UUG and a 69-bp ORF starting with AUA. Non-canonical start codons are only rarely found in plants, generally upstream from coding sequences (Laing *et al.*, 2015). If they are recognized by the translation initiation factors, the short nascent peptide or the process of a non-canonical translation initiation may influence mt protein synthesis. Future research, particularly comparative proteomic studies of F and H plants, is needed to test this possibility.

Our experimental design used siblings from the same maternal parent, so under the assumption of maternal inheritance, F and H individuals should all share the same mitochondrial haplotype. However, this assumption might be violated by processes known to affect plant mitochondrial genomes, including paternal leakage (Pearl *et al.*, 2009) and substoichiometric shifting (Janska *et al.*, 1998). We tested for potential effects of these processes in the following ways: (1) we cloned and sequenced the highly polymorphic gene *atp1* to rule out heteroplasmy and paternal leakage; and (2) we completed a *de novo* mt transcriptome assembly using mt-mapped RNA-seq reads and found no evidence of additional differentially expressed transcripts or novel, 50+ aa ORF-encoding chimeric proteins.

### Variation in DOC among individuals and within genes

Gene coverage values were consistent across all six individuals and had low standard deviations. The *rpl5* gene was the only exception, with 10-fold variation in DOC among individuals. This gene is absent in the mt genomes of *S. vulgaris* MTV and SD2, whereas a complete copy exists in KOV and S9L (Sloan *et al.*, 2012*b*). Highly variable expression descending to negligible values in some individuals may reflect the early (or even advanced) stages of a loss of function of this mt gene in *S. vulgaris* KOV. Despite a conserved coding sequence in the mt genome and conserved editing sites, *rpl5* may have been functionally replaced by a nuclear copy.

RNA-seq coverage across entire coding sequences was generally high. Sudden drops in coverage before the stop codon in four genes indicated the existence of transcripts without in-frame stop codons. Such transcripts of *ccmC* and *nad6* were documented previously in *A. thaliana* and *S. noctiflora* (Raczynska *et al.*, 2006, Forner *et al.*, 2007, Wu *et al.*, 2015*a*), while the absence of a stop codon in *atp1* transcripts was confirmed in *S. vulgaris* (Müller and Štorchová, 2013). In the case of *cob*, however, this is the first report of transcripts lacking a stop codon. In cases where the ends of a plant mitochondrial transcript precede the first in-frame stop codon, regions of prominent secondary structure may act as cleavage signals, such as the tRNA-like structures (t-elements) in *ccmC* and *nad6* (Forner *et al.*, 2007) and the hairpin-like structure in *atp1* (Müller and Štorchová, 2013). However, no such region was identified near the 5′ end of *cob*.

The *matR* gene exhibited a prominent drop in coverage (i.e. a ‘hole’ of about 600 bp) in the middle of the gene (Fig. 1C). The marginal parts of the *matR* coding sequence correspond to the RT and X domains. The internal drop in coverage may reflect the existence of two distinct mRNAs and the production of the two MatR domains as separate protein molecules. This scenario would be consistent with the recent finding in *Pelargonium* that *matR* has been transferred to the nucleus and split into two distinct genes (Grewe *et al.*, 2016). In addition, the transcription of the *matR* domain X from an internal promoter, which also directed the co-transcription with the *trans*-spliced *nad1* exon 5 and *nad5* exon 3, was described in wheat mitochondria (Farré and Araya, 1999). It is possible that an internal promoter also exists in *matR* of *S. vulgaris*. Alternatively, cleavage and degradation of the central portion of *matR* mRNA may be responsible for the drop in coverage. If the first domain of *matR* is translated from a short separate transcript not containing an entire ORF, this truncated mRNA represents the fifth transcript lacking a stop codon in the *S. vulgaris* KOV mitochondria.

Variation in DOC across the length of genes may also be affected by the abundance and accessibility of transcript fragments for RNA-seq. For example, there is evidence of higher abundance of small RNAs in some regions that are ‘protected’ by binding with pentatricopeptide repeat proteins (Ruwe and Schmitz-Linneweber, 2012;Zhelyazkova *et al.*, 2012).

### Variation in mt gene copy number and mtDNA content

Unlike the stable number of nuclear genes existing in a single cell, growing evidence indicates that not only the number of mitochondria per cell, but also genome copy number per mitochondrion may vary (Woloszynska *et al.*, 2006; Preuten *et al.*, 2010; Oldenburg and Bendich, 2015). In addition, the copy number of specific sequences and structural variants within mitochondrial genomes can change dramatically through a process known as substoichiometric shifting (Arrieta-Montiel and Mackenzie, 2011). Accordingly, we considered both DOC and gene copy number measured by qPCR to obtain realistic estimates of mt gene expression in *S. vulgaris*. The gene copy numbers relative to mt *rrn18* were close to 1 for *FSNR*, *nad9*, *nad9* 5′ UTR, and *matR*. Therefore, the observed variation in transcript levels must correspond to differences in rates of transcription and/or RNA degradation rather than shifts in DNA copy number. Minor deviations from 1 in estimates of relative gene copy number (e.g. *nad9*) may be due to a combination of statistical noise and partial degradation or replication of specific regions (Oldenburg and Bendich, 2015). These results are consistent with findings of variable DOC across the genome from mt sequencing efforts (Mower *et al.*, 2012).

In contrast, the copy number of the *Ss6* region was much higher and more variable than the rest of the genome. It was 2–6 times higher than mt *rrn18* and varied across tissues and individuals without any clear effect of gender. The *Ss6* region is located on chromosome 6 in the KOV genome and its copy number most likely reflects the chromosome copy number. Chromosome 6 is physically autonomous and exists in multimeric forms (Sloan *et al.*, 2012*b*). The multimers of various size and structure may be responsible for the detected variation in the chromosome copy number. There are no annotated features on chromosome 6; *Ss6* is the only transcribed region. It contains an imperfect inverted repeat that may underlie RNA secondary structure. *Ss6* is conserved across all four sequenced *S. vulgaris* mt genomes, and is always found on the smallest chromosome. Multiple autonomous chromosomes without annotated genes were discovered in the massive mt genome of *S. noctiflora* (Sloan *et al.*, 2012*a*). Some of these contained transcribed regions (Wu *et al.*, 2015*a*) and were conserved across populations (Wu *et al.*, 2015*b*). It is possible that the *Ss6* region on chromosome 6 of *S. vulgaris* may perform a function despite the absence of ORFs.

### High-frequency and partial RNA editing

In parallel with DOC, the extent of editing was very similar in F and H flower bud transcriptomes of *S. vulgaris*. The only highly differentially edited site was located in the *FSNR* region, which underlines its association with CMS in *S. vulgaris*. There was a strong correlation between the extent of editing and the impact of an editing event. Non-synonymous sites in protein-coding sequences and sites stabilizing the structure of group II introns were edited to the highest extent. In contrast, the vast majority of silent sites and edits in intergenic regions were only partially edited, which is consistent with findings in other plant mt genomes (Wu *et al.*, 2015*a*; Guo *et al.*, 2017). *Trans*-spliced intron sites, and adjacent exons, generally showed higher editing extents than those in or adjacent to *cis*-spliced introns (Fig. 5, Supplementary Data Set 1D). This may be attributable to *cis*-splicing reactions being generally faster than those in *trans*, affording the intron of the immature transcript less time in an accessible conformation. The set of 264 editing sites resulting in amino acid changes in *S. vulgaris* (Table 1) is very similar but not identical to the set of 264 non-synonymous edits found in mt genome of *S. latifolia* (Sloan *et al.*, 2010). Ten highly edited sites in *S. vulgaris* introns were found in at least one additional plant species, despite very limited information about mt intron editing in angiosperms. Evolutionary conservation indicates functional importance of the highly edited sites.

### The order of editing and splicing

RNA-seq data enables us to identify reads derived from unspliced transcripts and to assess their respective editing extents. The comparison of editing in spliced and unspliced RNAs makes it possible to estimate the order of editing and splicing, two critical steps in mt gene expression. RNA-seq also provides a higher resolution and much broader survey than targeted cDNA sequencing, used in previous studies of the editing/splicing order (Li-Pook-Than *et al.*, 2007; Farré *et al.*, 2012). At nearly every position where the estimation was possible, the majority of transcripts had been edited prior to splicing, which is in agreement with previous reports (Castandet *et al.*, 2010; Farré *et al.*, 2012). However, pre-splice editing extents varied from site to site within transcripts, presumably reflecting the relative rates of the individual editing reactions. Furthermore, pre-splice editing extents also varied between transcripts (Fig. 5), which may indicate differences in the relative rates of the splicing reactions. In contrast, the very limited variation in the extent of pre-splice editing among individuals emphasized the non-random character and precise control of the factors underlying editing and splicing. The rates of the editing and splicing reactions are complex, presumably influenced by the concentrations and efficiencies of their factors as well as the concentrations and accessibilities of the transcripts. Nevertheless, by comparing the extent of editing in pre- and post-splice transcripts, we gain perspective on the rates and sequence of these post-transcriptional modifications.

We found only three positions where pre-splice editing was zero or very low. The complete absence of editing in unspliced *nad7* exon 3 is intriguing. As the splicing event is downstream from an editing event, it is not expected to affect the editing factor’s target sequence, although the base at this position may influence editing factor binding. Previously, it has been suggested that sterics or relative reaction rates may explain this pattern (Li-Pook-Than *et al.*, 2007). Given the enormous predicted size of editing and binding factors (Fujii *et al.*, 2011*b*), steric interference might be expected to act at a larger distance. However, a block preventing the editing machinery from interacting with the regular *cis* recognition motif does not inhibit the splicing reaction. This suggests that the intron may block editing prior to splicing due to the secondary structure assumed by group II introns. Splicing occurs prior to editing in this site both in wheat (Li-Pook-Than *et al.*, 2007) and *S. vulgaris*, indicating that the order of post-transcriptional modifications is evolutionarily conserved.

The *nad7* exon 5 and *nad1* exon 4 edit sites are located 3 and 14 nt downstream of the splice junction, respectively. These sites apparently require splicing to create the *cis* motifs recognized by the editing complex. In contrast, the *nad4* exon 3 site, placed 6 nt downstream from a splice junction, is almost completely edited before splicing, which indicates that the *cis* motif in the unspliced transcript is sufficient for editing. Interestingly, the same site was reported to be edited only after splicing in wheat (Li-Pook-Than *et al.*, 2007). The discrepancy in the editing/splicing order between wheat and *S. vulgaris* suggests the utilization of different *cis* recognition motifs for editing in the two species: unspliced sequence in *S. vulgaris* and spliced sequence in wheat. Distinct motifs may require different recognition proteins, or the *S. vulgaris* recognition factors may be capable of recognizing both pre- and post-spliced sequences.

A detailed analysis of the editing and editing/splicing order in mt transcriptomes from *S. vulgaris* showed a general uniformity and found only one marked difference between F and H plants – the editing site within the *FSNR* region, which was also differentially expressed between the genders. Although the possibility that *FSNR* codes for an extremely short peptide cannot be completely excluded, the most parsimonious conclusion is that *FSNR* RNA is non-coding. Therefore, *FSNR* represents an attractive candidate for future functional validation studies to assess whether non-coding RNAs could play a causal role in CMS.

## Supplementary data

Supplementary data are available at *JXB* online.

Fig. S1. RNA-seq read coverages for genes producing transcripts without stop codons.

Fig. S2. Least-squares estimates of mean RNA editing rates for females and hermaphrodites by genomic location.

Table S1. RNA editing sites in the mitochondrial introns of *S. vulgaris* and four additional angiosperm species.

Method S1. Bioinformatic analyses.

Data Set S1. Depth of coverage for genes, ORFs, and intergenic regions, editing sites, editing in spliced and unspliced transcripts, and primers used in qPCR.

## Supplementary Material

supplementary_figures_S1-S2+protocol_S1+Table_S1

supplementary_dataset_S1
