# Supplementary material for: Non-coding RNA may be associated with cytoplasmic male sterility in Silene vulgaris
Source: J Exp Bot. 2017 Mar 29;68(7):1599–612. doi: 10.1093/jxb/erx057 (PMC5444436; doi:10.1093/jxb/erx057)
Supplement: supplementary_figures_S1-S2+protocol_S1+Table_S1 [file erx057_suppl_supplementary_figures_S1-S2+protocol_S1+Table_S1.pdf]

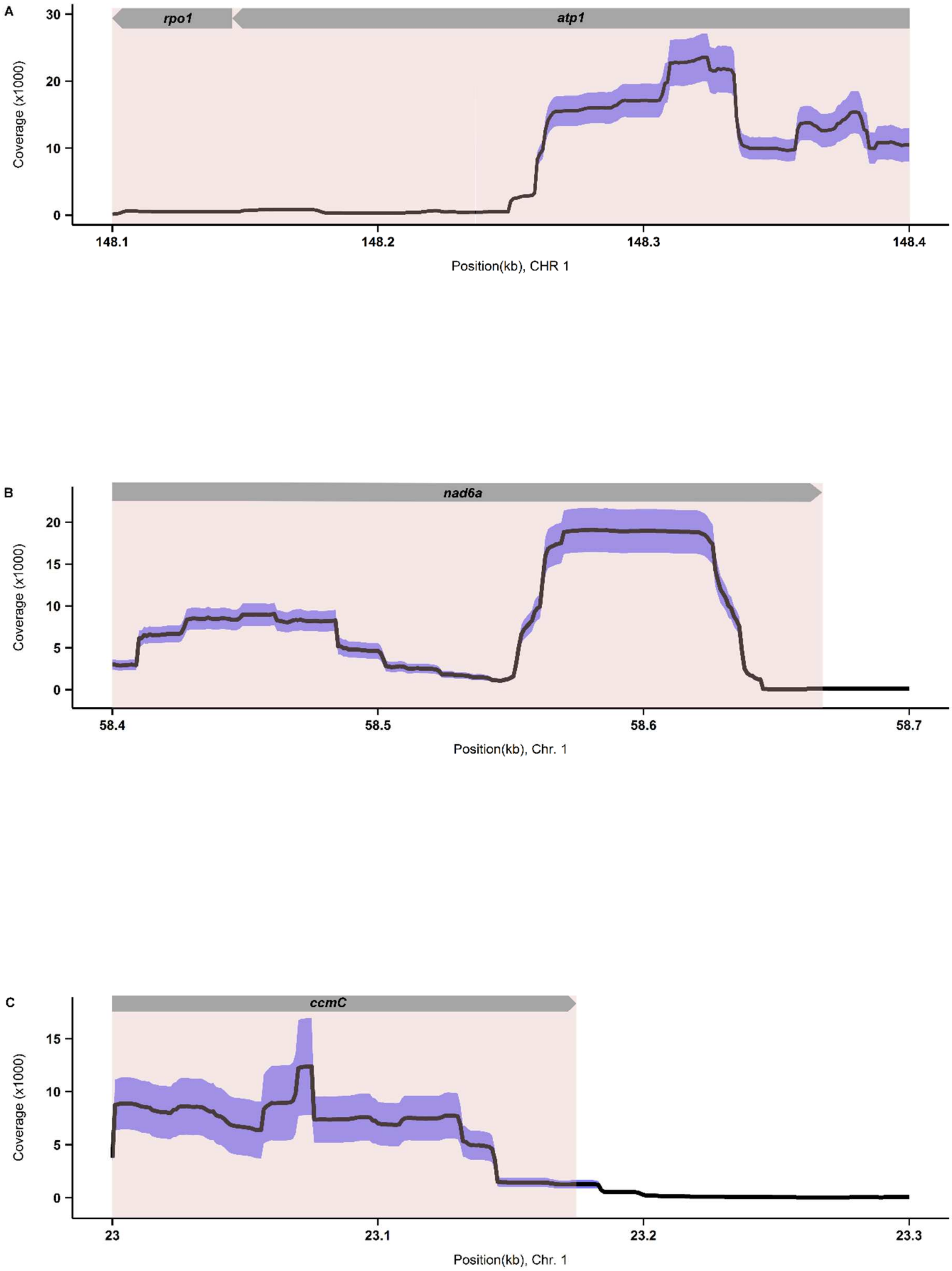

Supplemental Figure 1. RNA-seq read coverages for genes producing transcripts without stop codons. Mean DOC across individuals are given by the plots' lines, while standard deviations in DOC are given by the blue bands. Values are for all six individuals in A-D, and split into female (n=3) and hermaphrodite (n=3) for E. **A.** *atp1* **B.** *nad6* **C.** *ccmC*.

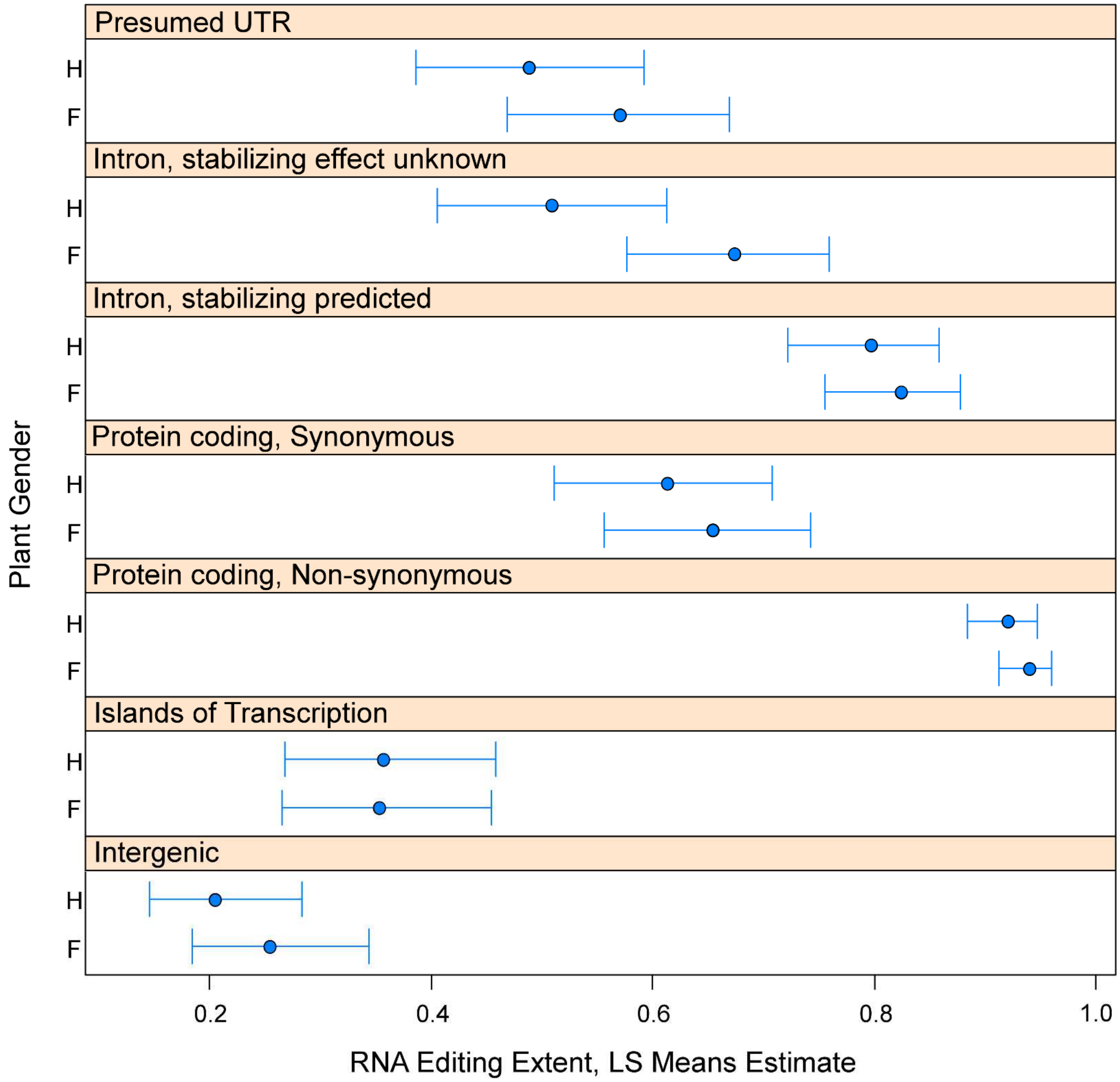

Supplemental Figure 2. Least-squares estimates of mean RNA editing rates, ± 99% confidence intervals, for female (F, n=3) and hermaphrodite (H, n=3), by genomic location.

Method S1. Bioinformatic procedures.

Read mapping

Initial alignment was performed using GSNAP v. 2014-12-23 (Wu and Nacu, 2010) in paired-end mode using known splice junctions (Sloan et al., 2012) and default settings otherwise. Alignments were strand-separated using the view function in SAMtools v. 1.1 (Li et al., 2009), filtering by SAM flags according to read-pair orientation. First reads in forward orientation corresponded to positive-strand transcription, while those in reverse orientation corresponded to the negative strand. The opposite rules applied for second reads. An initial round of variant discovery using the HaplotypeCaller module in GATK 3.4 (McKenna et al., 2010) was performed separately on the plus- and minus-strand alignments, with default settings except that the minimum call and emit thresholds were set to 20. Only variant sites that could be attributed to canonical mitochondrial RNA editing were retained (C-to-T variants from the plus-strand alignment, G-to-A from the minus strand). High-identity (>99%) repeat regions longer than 300 bp and regions with high plastid similarity, identified through mitochondrion-by-mitochondrion and mitochondrion-by-plastid genome blast searches, were ignored during variant calling. Variant sites with a GATK quality score of 1000 or lower were discarded, reducing false positive rates at the expense of excluding some legitimate editing sites, particularly partial and intergenic sites. This threshold was chosen based on manual inspection of low quality variant sites not fitting the canonical editing pattern. Filtered variant sites were compared with raw variant sites to estimate false positive rates. The set of filtered, high quality variant sites and known splice junctions were then used to generate splice- and editing-tolerant alignments with GSNAP using the same settings as before, but including the set of high quality variant sites in “SNP-tolerant” mode. The GATK base recalibrator module was used with the set of high quality variant sites and the set of splice- and editing-tolerant alignments to recalibrate base quality scores. These recalibrated alignments were used for a final round of variant discovery. High quality (GATK haplotype caller score of >1000) variant sites were retained for final analysis. The complete set of variant sites from the final variant discovery, regardless of quality score, was retained for select analyses, particularly comparison with edit sites reported in other studies (Sloan et al., 2010, Bentolila et al., 2013, Wu et al., 2015a).

Lastly, a set of final splice- and editing-tolerant alignments using the final, filtered set of editing sites and known splice junctions was generated as before and used for unbiased editing rate estimation as well as transcript abundance estimation.

Defining islands of transcription

In order to objectively identify high coverage intergenic features, we first gathered mt-genome-wide information about distributions of alignment depth of coverage. We used the makewindows function in bedtools v 2.24.0 (Quinlan and Hall, 2010) to generate 200-bp sliding window “tiles” across the mt genome at 100-bp intervals. We then used the bedtools intersect function to classify these windows as genic, intronic, ORF, or

intergenic. A window with any overlap with an exon was classified as genic. Any remaining windows that overlapped introns were classified as intronic. In the case of trans-spliced introns, the boundaries were estimated, so it is possible that additional windows adjacent to our conservative trans-spliced intron boundaries actually contained intronic sequence. Any remaining windows that overlapped with ORFs with a minimum length of 300 bp were classified as such. All remaining windows were classified as intergenic. As the true trans-spliced intron boundaries are unknown and transcription from all transcribed features may continue beyond their defined boundaries, we calculated the distance between intergenic features and the nearest annotation. Intergenic tiles were further subdivided into "isolated" or "adjacent to another feature" categories, depending on whether or not they were within 500 bp of an annotated feature. Normalized depth of coverage, based on the total number of mt-mapped reads for each sample, was calculated for all windows using the bedtools coverage function and averaged across samples. Intergenic features with average depth of coverage exceeding 1020× were classified as "islands of transcription." This threshold was chosen as the depth of coverage at which genic features had a higher probability density than intergenic features. All windows overlapping, adjoining, or within 100 bp of each other were merged into a single "island" using the merge function in bedtools.

Transcript abundance estimation

Transcript abundance estimation was performed using the bedtools coverage function and a custom AWK script. Per-base coverage was calculated and averaged over the length of annotated features for each sample. Coverages were then normalized as TPM (Wagner et al. 2012). Average and standard deviation TPM values were calculated for both sexes and both strands for all features of interest.

Spliced and unspliced read parsing and estimate of RNA editing rates

Aligned read pairs derived from unspliced RNAs were identified on the basis of overlap with both genic and intronic annotations. Parsing was performed using the SAMtools view function and the bedtools intersect module. Aligned read pairs derived from spliced RNAs were identified based on their SAM format transcript length (TLEN) parameter and isolated using the SAMtools view function and an AWK script. Read pairs with TLEN of 1 kb or longer were designated as having undergone at least one splicing event. These reads were well outside the range of most aligned reads' TLEN values (a mean length of 196 bp, and a standard deviation of 36 bp were calculated from the population of all mt-aligned reads with TLEN < 1 kb).

To calculate rates of RNA editing, the SAMtools mpileup function was supplied with the list of identified editing sites. Editing extent was calculated as the count of Ts divided by the sum of Cs and Ts. These calculations were performed to determine the overall editing rate and to distinguish between the extent of editing in transcripts before and after intron splicing for all sites where pre- and post-splice reads could be identified in each sample.

Statistical analyses

Statistical analyses were performed using R v 3.1.1. The glmer package was used to conduct ANOVAs and generate LSMEANS estimates of editing extent for each gender and category of editing site. Editing extent was modeled as a binomial variable, with genomic region, sex, and their interaction as categorical factors and individual plant included as a random factor. Plots were generated using the ggplot2 and base libraries.

References

Bentolila S, Oh J, Hanson MR, Bukowski R. 2013. Comprehensive high-resolution Analysis of the role of an Arabidopsis gene family in RNA editing. PLoS Genet **9**, e1003584.

Li H, Handsaker B, Wysoker A, Fennell T, Ruan J, Homer N, Marth G, Abecasis G, Durbin R. 2009. Subgroup 1000 Genome Project Data Processing: The Sequence Alignment/Map format and SAMtools. Bioinformatics **25**, 2078–2079.

McKenna A, Hanna M, Banks E, *et al.* 2010. The Genome Analysis Toolkit: A MapReduce framework for analyzing next-generation DNA sequencing data. Genome Research **20**, 1297-1303.

Quinlan AR, Hall IM. 2010. BEDTools: a flexible suite of utilities for comparing genomic features. Bioinformatics **26**, 841-842.

Sloan DB, **Mueller K**, **McCauley D.E., Taylor DR, Štorchová H.** 2012. Intraspecific variation in mitochondrial genome sequence, structure, and gene content in *Silene vulgaris*, an angiosperm with pervasive cytoplasmic male sterility. New Phytologist **196,** 1228-1239.

Wagner GP, Kin K, Lynch VJ. 2012. Measurement of mRNA abundance using RNA-seq data: RPKM measure is inconsistent among samples. Theory in Biosciences **131**, 281-285.

Wu TD, Nacu S. 2010. Fast and SNP-tolerant detection of complex variants and splicing in short reads. Bioinformatics **26**, 873-881.

Table S1. RNA editing sites in the mitochondrial introns of *S. vulgaris* and four additional angiosperm species.

CHR	Position	Feature	*Silene vulgaris*	H avg Extent	F avg Extent	diff/ avg	DOC avg	domain	*Silene noctiflora*	*Oenothera*	Tobacco	Wheat
1	265769	*nad1* intron trans1.b	C					VI stem	C	C	C	**C > T** [7]
1	265770	*nad1* intron trans1.b	C					VI stem	C	**C < T** [1]	C	C
1	265774	*nad1* intron trans1.b	C					VI stem	C	**C < T** [1]	C	Deletion
1	264377	*nad1* intron 2.3	C > T	0.179	0.242	0.30	157	IV loop	A	C	deletion	A
1	263539	*nad1* intron trans3.4a	**C > T**	**0.564**	**0.739**	0.27	130	I	C	not known	**C > T** [6]	C, no evidence for editing
1	102762	*nad1* intron trans3.4b	**C > T**	**0.933**	**0.954**	0.02	8	VI stem	C	not known	**C > T** [6]	**C > T** [7]
1	103457	*nad1* intron trans4.5a	**C > T**	**0.920**	**0.975**	0.06	47	IV loop	C	not known	C	C, no evidence for editing
1	184560	*nad1* intron trans4.5b	T					VI stem	T	not known	**C > T** [6]	**C > T** [7]
1	247114	*nad2* intron1.2	C > T	0.300	0.400	0.28	112	IV loop	deletion	deletion	deletion	deletion
1	247475	*nad2* intron1.2	**C > T**	**0.717**	**0.725**	0.01	275	IV stem	**T**	**C > T** [2]	**C > T** [6]	C, no evidence for editing
1	247935	*nad2* intron trans2.3a	**C > T**	**0.923**	**0.900**	0.03	148	I	**C > T** [9]	**C > T** [3]	**C > T** [6]	**C > T** [7]
1	174746	*nad2* intron trans2.3b	**C > T**	**0.532**	**0.623**	0.16	131	IV stem	C	**C > T** [3]	C	C, no evidence for editing
1	176213	*nad2* intron3.4	C > T	0.244	0.450	0.59	119	I	C	not known	C	C, no evidence for editing
1	177450	nad2 intron4.5	C > T	0.439	0.621	0.34	90	I	C	not known	C	C, no evidence for editing
4	8400	*nad4* intron1.2	**C > T**	**0.829**	**0.911**	0.09	51	I	C	not known	T	**C > T** [7]
4	3608	*nad4* intron2.3	T					VI stem	T	not known	**C > T** [6]	**C > T** [7]
1	205741	*nad5* intron1.2	**C > T**	**0.620**	**0.810**	0.26	41	VI bulge	C	**C > T** [4]	C	T
1	205736	*nad5* intron1.2	T					VI stem	T	T	**C > T** [6]	T
1	204246	*nad5* intron trans2.3a	**C > T**	**0.750**	**0.787**	0.05	128	I	**C > T** [9]	C	**C > T** [6]	T
1	196607	*nad5* intron trans2.3b	T					VI stem	T	**C > T** [5]	**C > T** [6]	**C > T** [7]
1	16591	*nad7* intron2.3	**C > T**	**0.850**	**0.913**	0.07	100	VI stem	T	not known	**C > T** [6]	T
1	15126	*nad7* intron 3.4	**C > T**	**0.753**	**0.913**	0.19	105	V stem	deletion	not known	deletion	C
1	15125	*nad7* intron 3.4	T					V stem	deletion	not known	deletion	**C > T** [8]
1	13187	*nad7* intron 4.5	T					V stem	T	not known	C	**C > T** [8]
1	13203	*nad7* intron 4.5	T					V stem	T		**C > T** [6]	T
1	126651	*ccmFc* intron1.2	T					V stem	T		**C > T** [6]	**C > T** [7]

H**,** hermaphrodite; F, female; avg, average; DOC, depth of coverage

1) Wissinger et al. 1991 **2**) Lippok et al. 1994 **3**) Binder et al. 1992 **4**) Knoop et al. 1991 **5**) Knoop et al. 1997 **6**) Grimes et al. 2015
7) Li-Pook-Than et al. 2007 **8**) Carillo and Bonen 1997 **9**) Wu et al. 2015
